# Guillain–Barré Syndrome Likely due to Relapsing Hepatitis A

**DOI:** 10.1155/2021/5570027

**Published:** 2021-05-26

**Authors:** Edgar Blecker, Maryam Ehtsham

**Affiliations:** Department of Internal Medicine, Methodist Dallas Medical Center, Dallas, TX 75203, USA

## Abstract

Guillain-Barré syndrome (GBS) is an immune-mediated disease of the peripheral nervous system that can be caused by various bacterial and virologic agents. The disease is characterized by progressive muscle weakness and paralysis. Rarely, GBS is preceded by an acute infection with hepatitis A. Here, we present the case of a 53-year-old woman who presented with progressively worsening motor weakness in the distal extremities. She reported a preceding gastrointestinal infection with nausea, vomiting, and diarrhea two weeks prior to her presentation to the emergency department. She was noted to have elevated serum transaminase levels and hepatitis A IgM and IgG antibodies signifying likely relapsing hepatitis A. She was later diagnosed with GBS on the basis of clinical findings and albuminocytologic dissociation in the cerebrospinal fluid. She was treated with intravenous immunoglobulin with subsequent improvement in her strength.

## 1. Introduction

Guillain–Barré syndrome (GBS) is a peripheral demyelinating disorder often preceded by a gastrointestinal infection. The causative infectious agent is usually bacterial or viral with the most common causes being *Campylobacter*, influenza virus, Ebstein–Barr virus, and cytomegalovirus infections. GBS is rarely caused by hepatitis A.

## 2. Case Presentation

The patient was a 53-year-old woman with a past medical history of hypothyroidism and hyperlipidemia who presented to the emergency department (ED) with worsening bilateral upper and lower extremity weakness for three days. Two weeks prior to her presentation, she reported fever, diarrhea, nausea, vomiting, and pain on the right side of the abdomen. She had presented to the ED one week prior due to abdominal pain with a mild elevation in her liver enzymes. No acute pathology was discovered, and she was told to follow-up with her primary care physician (PCP). She then presented to an urgent care facility in which she was prescribed ciprofloxacin with eventual improvement in her diarrhea and vomiting. Three days prior to arrival, she began to note numbness and weakness of her fingers and toes. Her weakness was most noticeable in the distal aspects of her extremities, and she experienced some difficulty ambulating and a prominent decrease in her grip strength. This prompted her to schedule an appointment with her PCP. She followed up with her PCP, who was concerned for demyelinating disease. The PCP urged her to present to the ED for further evaluation. Despite not having any respiratory symptoms or fever, her PCP tested her for COVID-19 and influenza given her abdominal pain and diarrhea. Both tests returned negative.

Further history revealed the patient had reported a prior presentation to the ED roughly 10 months earlier for right upper quadrant abdominal pain. She was told she had elevated liver enzymes at that visit and was told to follow-up with her PCP. Her PCP had tested her for hepatitis C, which was negative, and she eventually had resolution of her clinical symptoms. She denied any recent travel, fevers, or shortness of breath. She had mostly been indoors for the past two months, without reported sick contacts.

Physical examination upon presentation to the ED was notable for 4/5 strength in the bilateral upper extremities with flexion and extension of the elbow and wrist. She had decreased grip strength bilaterally. Bilateral lower extremities had 3/5 strength with flexion and extension of the hip and knee as well as decreased dorsiflexion and plantar flexion of both feet. She had decreased sensation in her lower extremities with absent reflexes throughout. Initial labs were significant for a normal TSH, free T4, and a mild elevation of her liver enzymes: aspartate transaminase (AST) of 40 U/L (normal range: 8–48 U/L), alanine transaminase (ALT) of 46 U/L (normal range: 7–55 U/L), and alkaline phosphatase (ALP) of 138 U/L (normal range: 40–129 U/L). A hepatitis panel and computed tomography (CT) scan abdomen/pelvis were ordered to investigate her transaminitis. She was positive for hepatitis A IgG and IgM antibodies with no acute pathological process noted on the CT scan.

Initial differential diagnosis included GBS versus a compressive myelopathy of the spine. Neurology was consulted with recommendations to perform a lumbar puncture and obtain further imaging of the brain and spine. A CT scan of the head was without acute abnormalities, and magnetic resonance imaging of the cervical, thoracic, and lumbar spine was negative for compressive myelopathy or acute abnormalities. A lumbar puncture was performed with an elevated total protein of 72 mg/dL (normal range: 15–60 mg/dL) and white blood cell count of 5 cells/mm^3^ (normal range: 0–8/mm^3^) suggesting albuminocytologic dissociation. The patient was diagnosed with GBS and started on intravenous immunoglobulin (IVIG) for five days. She had noticeable improvement in her distal extremity weakness following the first few doses of IVIG and was able to ambulate on her own by the end of her treatment. She was able to discharge following completion of her immunotherapy.

## 3. Discussion

GBS pathogenesis occurs through molecular mimicry in which the immune system is activated by an infection leading to autoimmune injury of peripheral nerve components. This immune response leads to the progressive neurological findings seen in GBS and its variants. Most commonly, the etiologic agent is either bacterial or viral with *Campylobacter jejuni* being the most common cause, followed by cytomegalovirus, Epstein–Barr virus, and influenza [1].

GBS due to hepatitis A is rare and has been reported in a few case reports with relatively good outcomes [2]. Coinfection with bacterial or viral agents has been reported, and thus, evaluation for concomitant infection should be conducted [3]. The patient in the case was ruled out for influenza and COVID-19. However, campylobacter, EBV, and CMV were not evaluated for.

Diagnosis of GBS typically includes the classic findings of symmetric muscular weakness of the distal extremities and absent or decreased reflexes, with a monophasic disease course and nadir of weakness between 12 hours and 28 days from the start of therapy [4]. CSF fluid showing albuminocytologic dissociation and electrodiagnostic studies can support the diagnosis and further delineate between the associated variants of GBS. Our patient had the classic clinical findings with a rapid improvement in her weakness following treatment with IVIG and albuminocytologic dissociation to support the diagnosis. Given high probability for diagnosis and delay in having staffing available to perform electrodiagnostic studies, this was not performed on our patient. Treatment for GBS centers around either plasma exchange or IVIG [5]. Patients are carefully monitored for respiratory symptoms and autonomic disturbances including arrhythmias, which are commonly reported.

Hepatitis A is a known cause of acute liver injury or liver failure in the United States. The clinical course of hepatitis A is usually self-resolving with most patients having a complete recovery. Serum hepatitis A antibodies can be helpful to determine whether there is an active or past infection. Patients will typically develop IgM antibodies to hepatitis A within two to three weeks of the initial infection. The presence of IgM antibodies indicates an active infection; circulating IgM antibodies can persist for up to six months. Roughly one to two weeks after developing serum IgM antibodies, IgG antibodies will develop and will persist throughout life. An elevated IgG antibody level indicates a past infection or prior vaccination [6].

Following acute infection, hepatitis A patients develop immunity to further infection. Rarely, a small group of patients (around 10%) will develop a relapsing course. Relapsing hepatitis A is most common between 30 and 90 days after acute infection but can be seen up to 12 months after [2]. Relapse is characterized by a preceding infection with a resolution of clinical and laboratory findings, followed by relapse weeks to months after the original infection. Serum IgM antibodies will often remain positive throughout relapse. Relapse is often milder than the acute infection, with aminotransferases sometimes rising above 1,000 IU/dL. Liver enzymes during relapse tend to show a cholestatic pattern. Extrahepatic manifestations of hepatitis A are more common during relapse. These manifestations include a pruritic rash and arthralgia. Relapsing infection has also been shown to cause immunological disturbances with late-onset arthritis, purpura, vasculitis, and myocarditis among others [7, 8]. An association with relapsing hepatitis A and GBS has not been demonstrated in the literature.

Although our patient was diagnosed with an acute hepatitis A infection on the basis of her positive IgM antibodies and mild transaminitis, it is difficult to determine the timing of the initial infection. She reported a similar episode of right upper quadrant abdominal pain roughly 10 months prior. Her labs at her PCP visit at that time showed a mild elevation of her liver enzymes as well. Unfortunately, hepatitis A was not tested for during that presentation. During her hospitalization, she had both positive IgM and IgG antibodies for hepatitis A, indicating an acute infection within the past six months. It is presumed that her nausea, vomiting, and diarrhea two weeks before presentation was due to a relapse of her hepatitis A given her serologies, although other causes of diarrhea were not ruled out as her symptoms had largely resolved at the time of presentation.

In conclusion, GBS should be investigated for in any patient presenting with symmetric distal extremity weakness. History is important to determine the likely causative agent. Although hepatitis A is not classically associated with GBS, it has been shown to have an association. Suspicion should be high in any patient presenting with elevated liver enzymes and can be confirmed with antibody testing for hepatitis A.

## Data Availability

Data were adapted from patient's clinical hospital course. Underlying data can be obtained, if needed, from patient's hospital chart, with approval from Methodist Dallas Medical Center.
